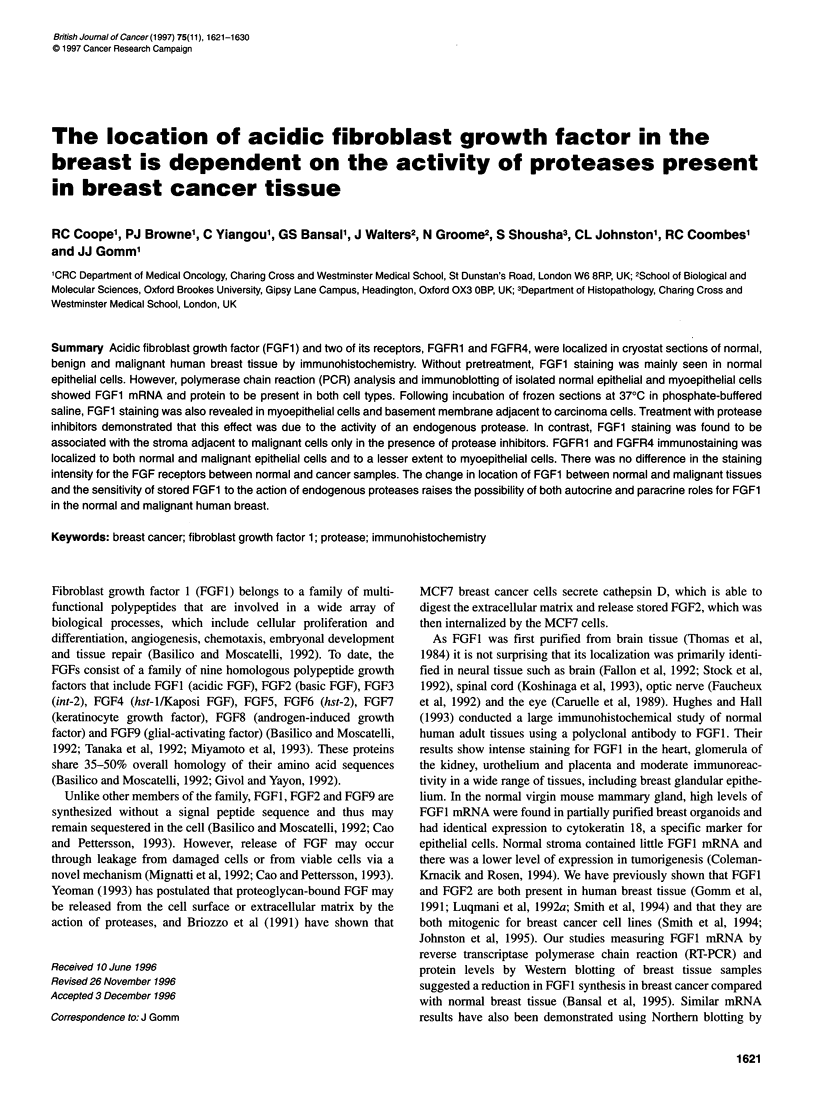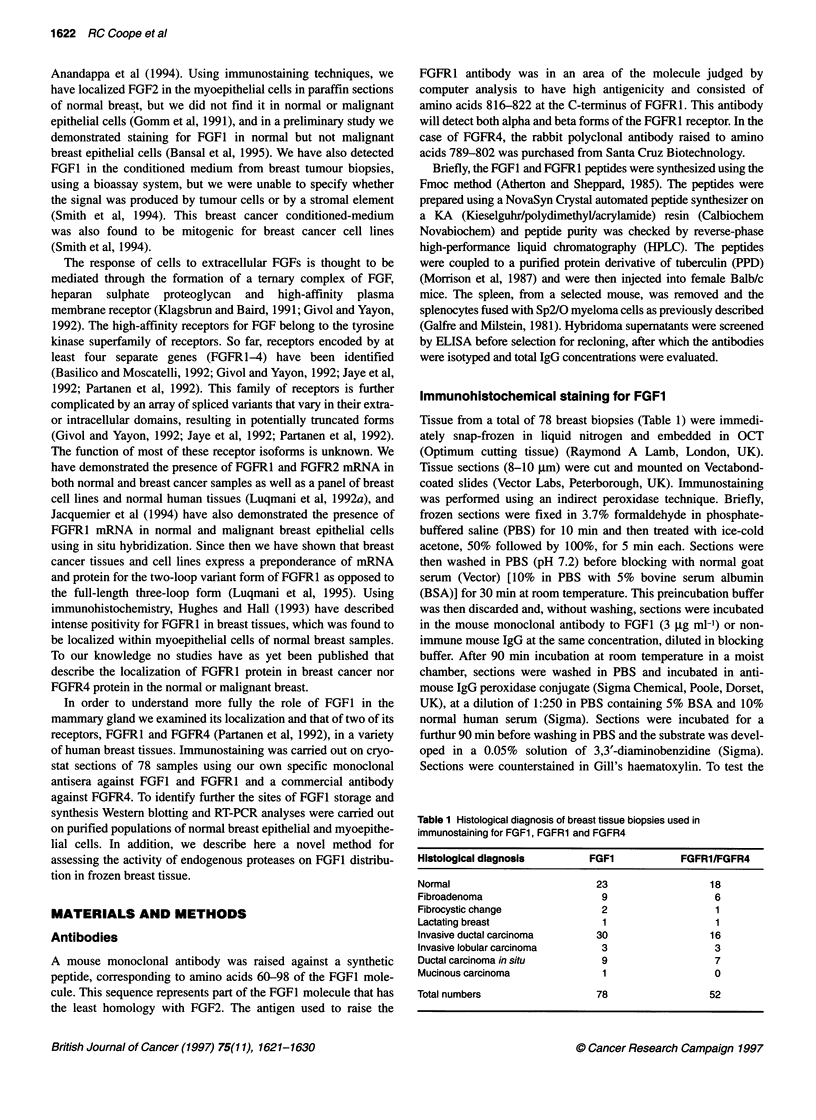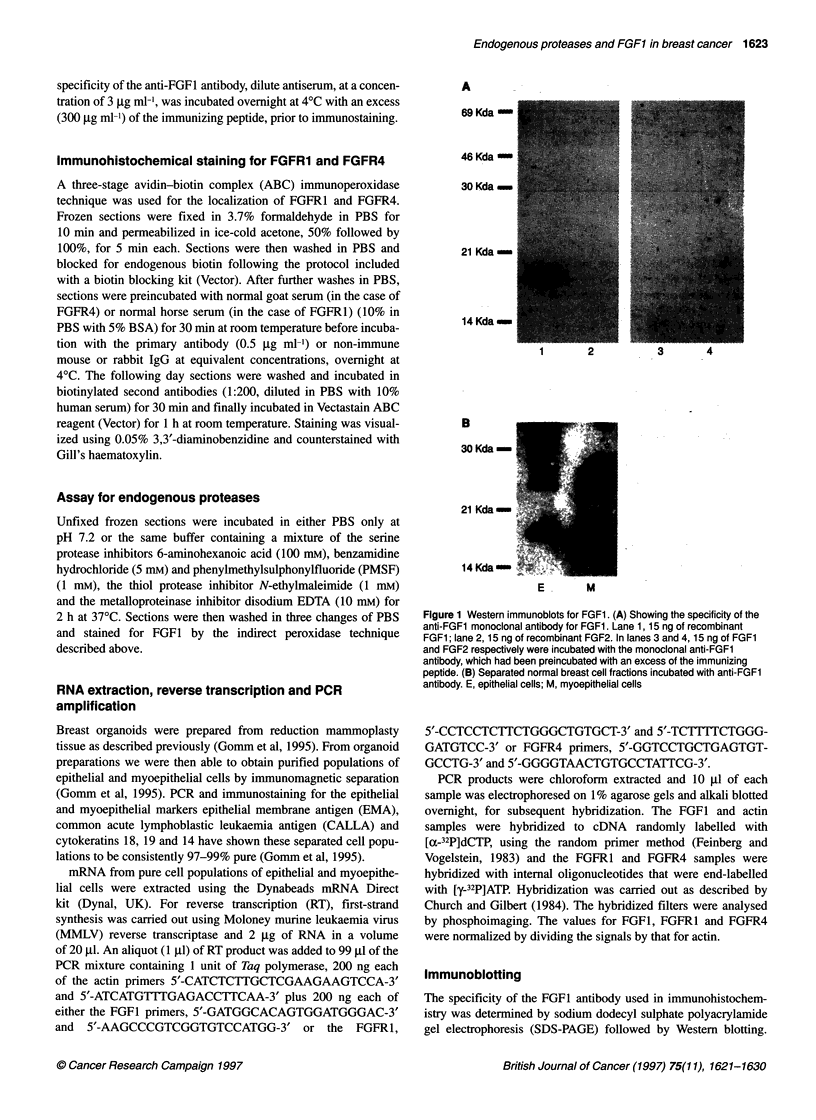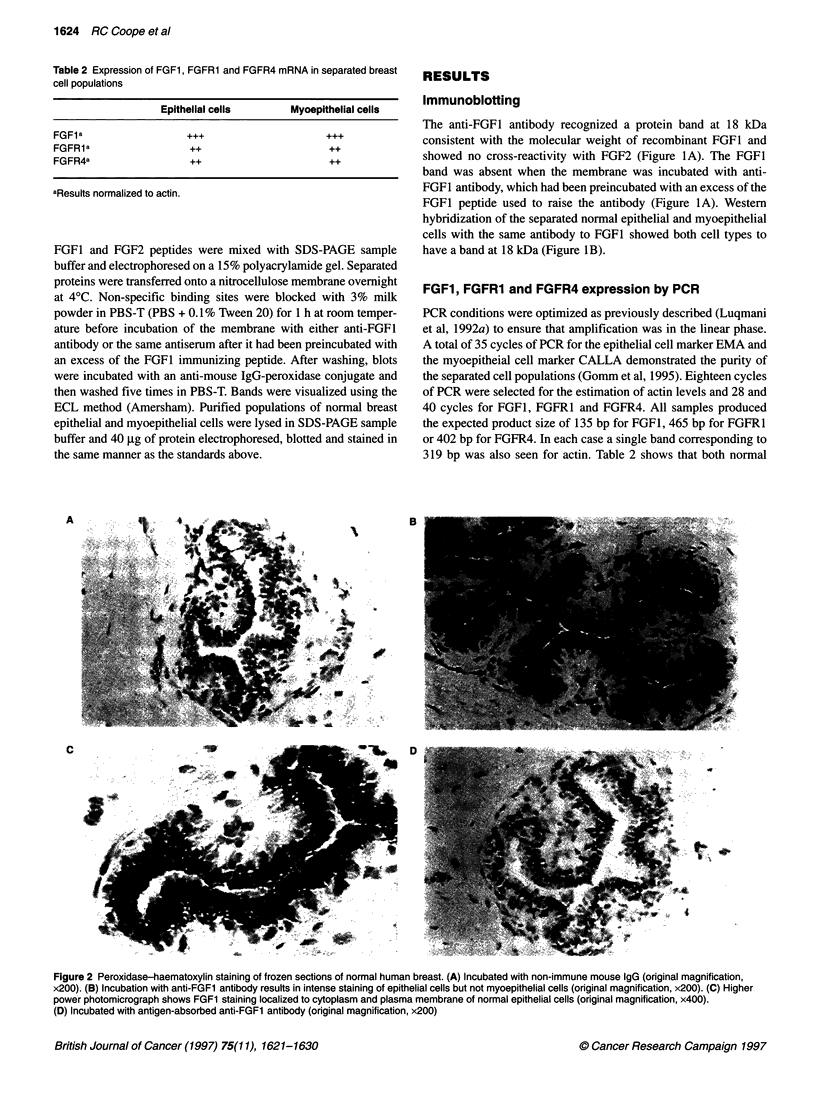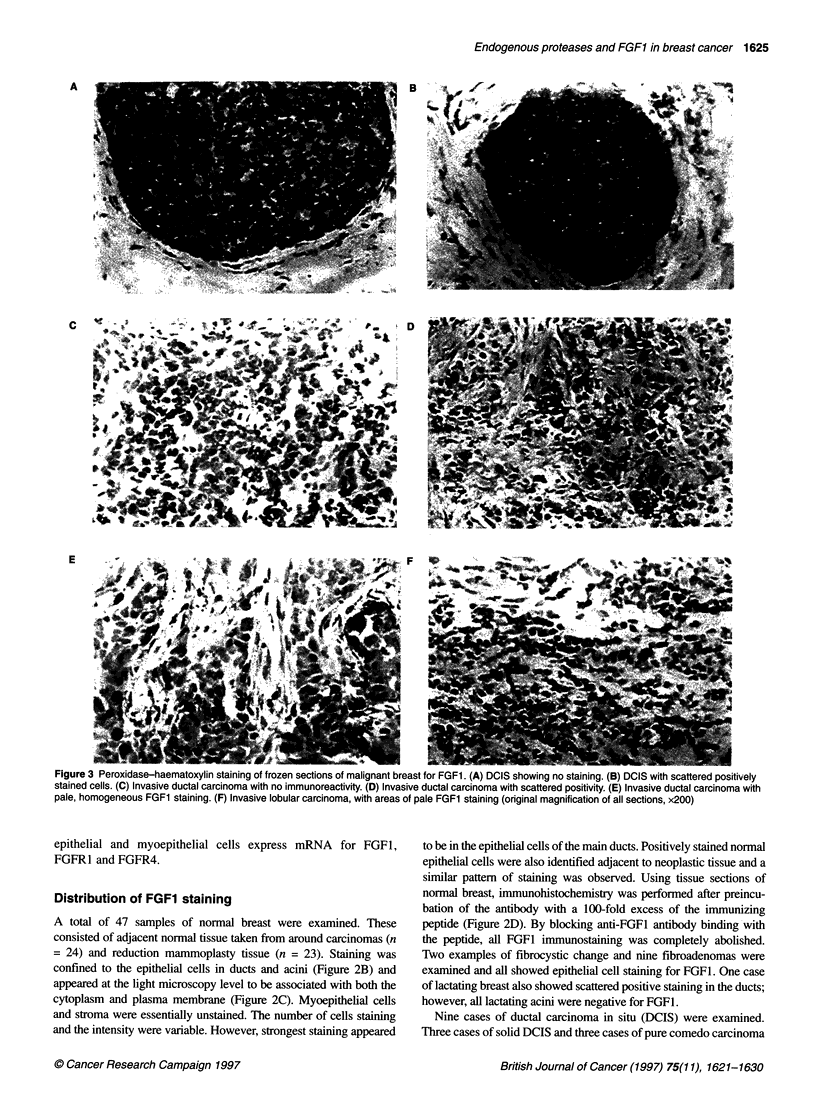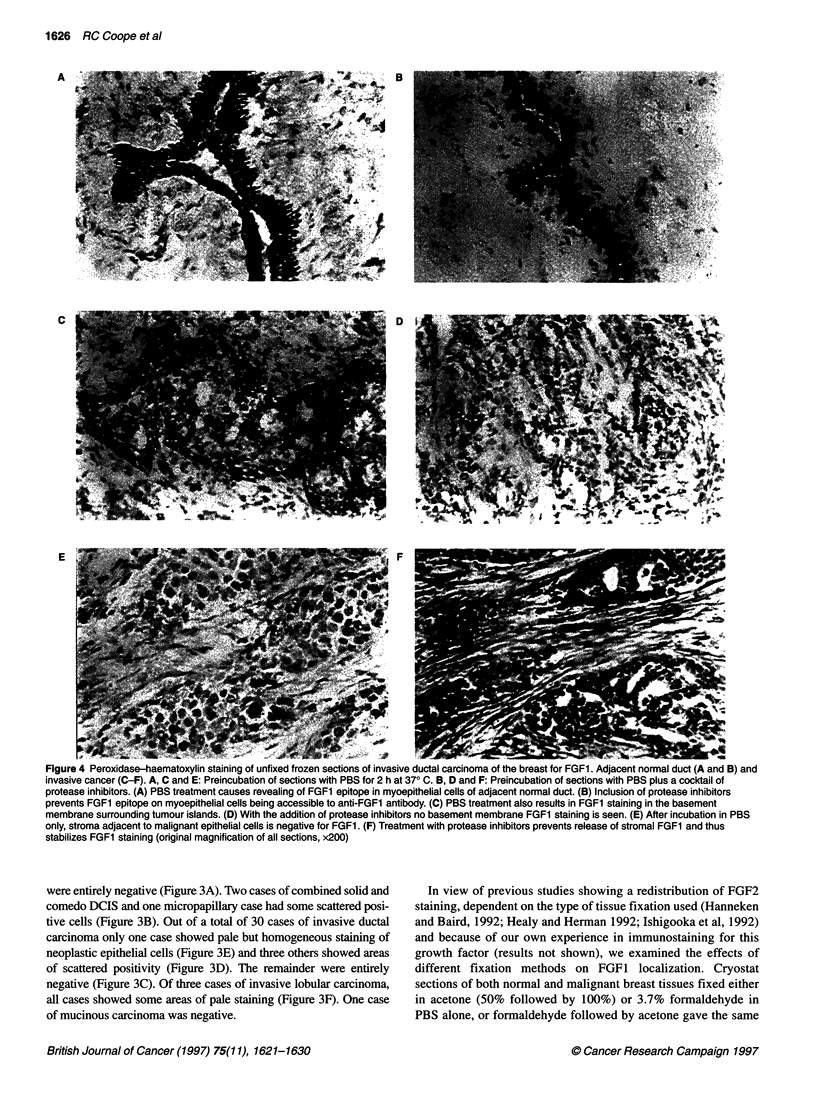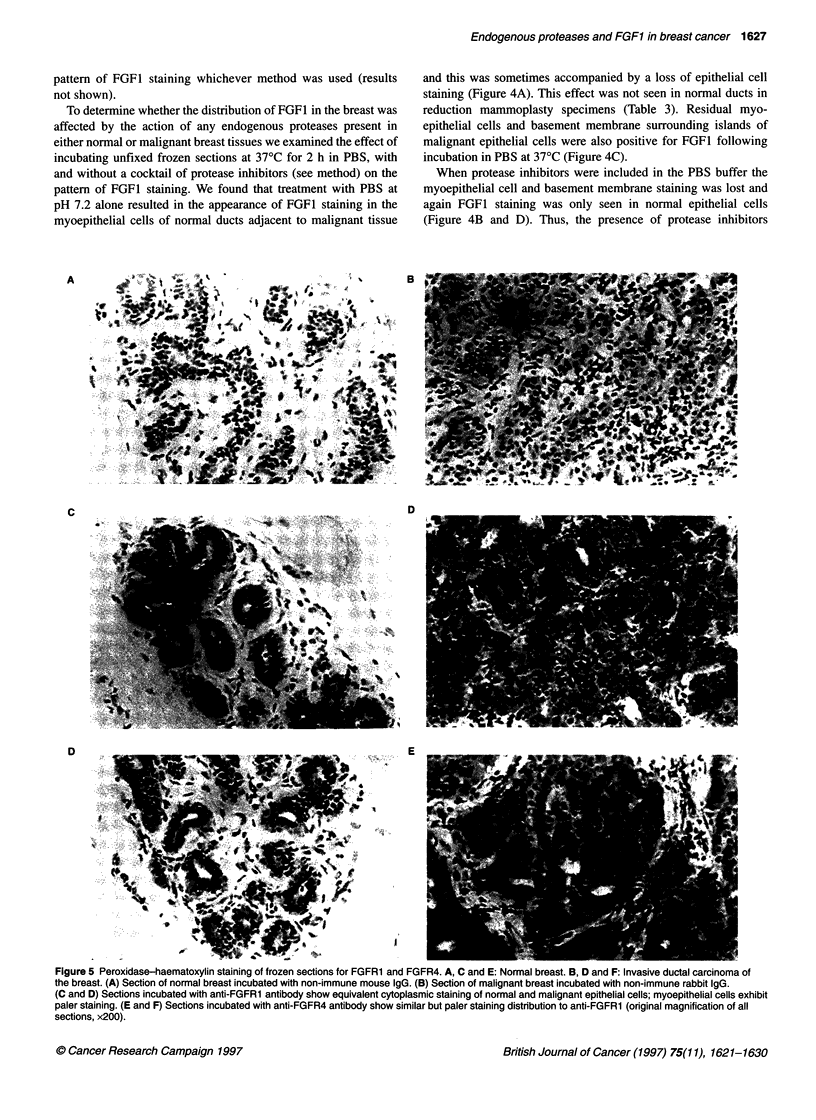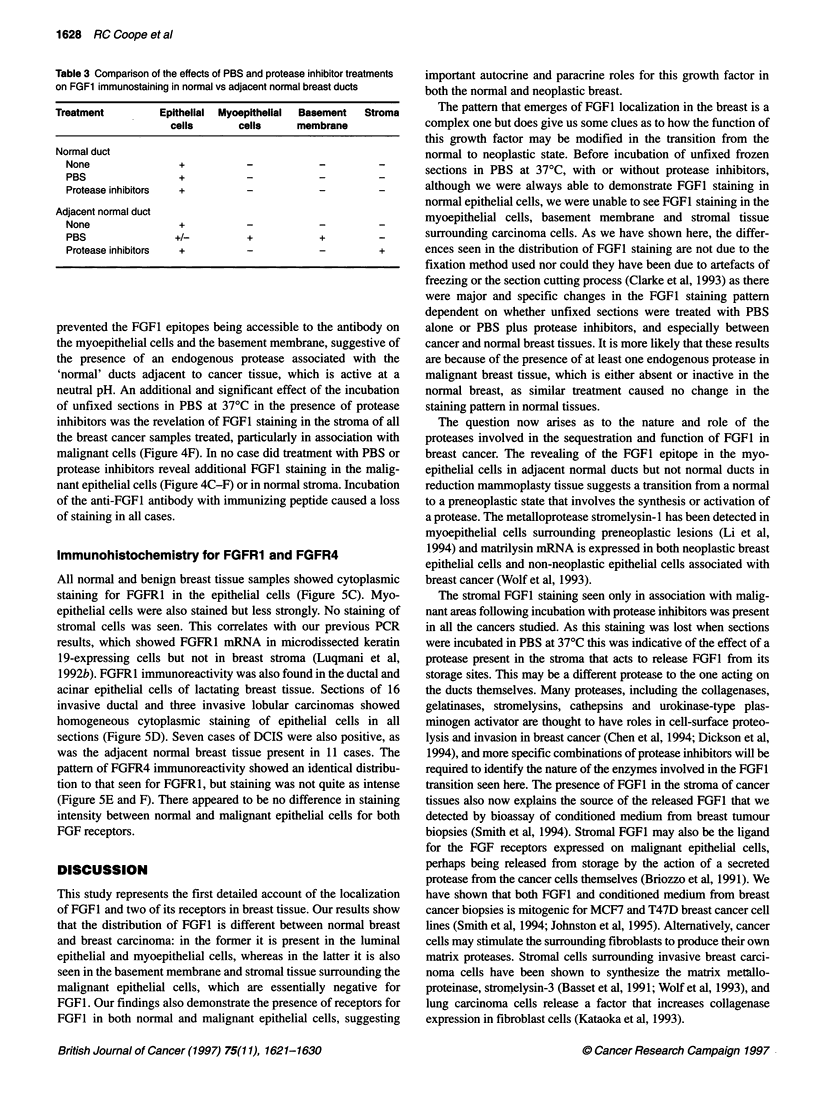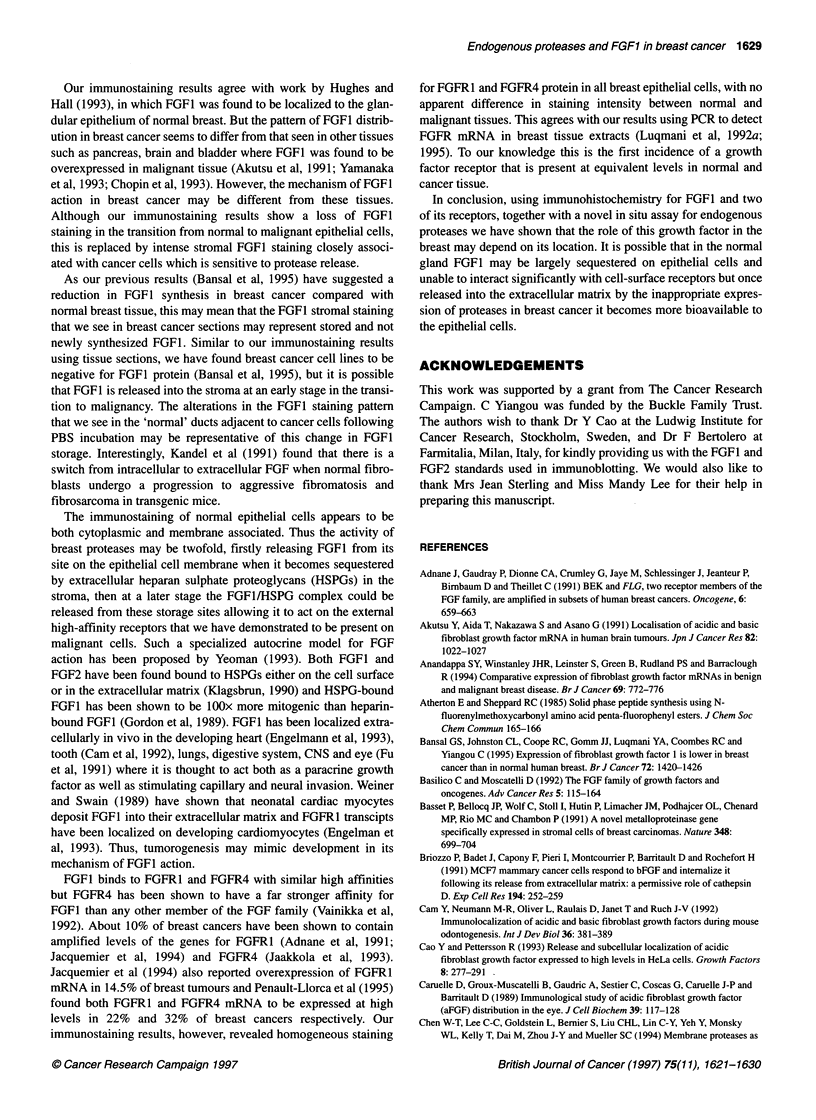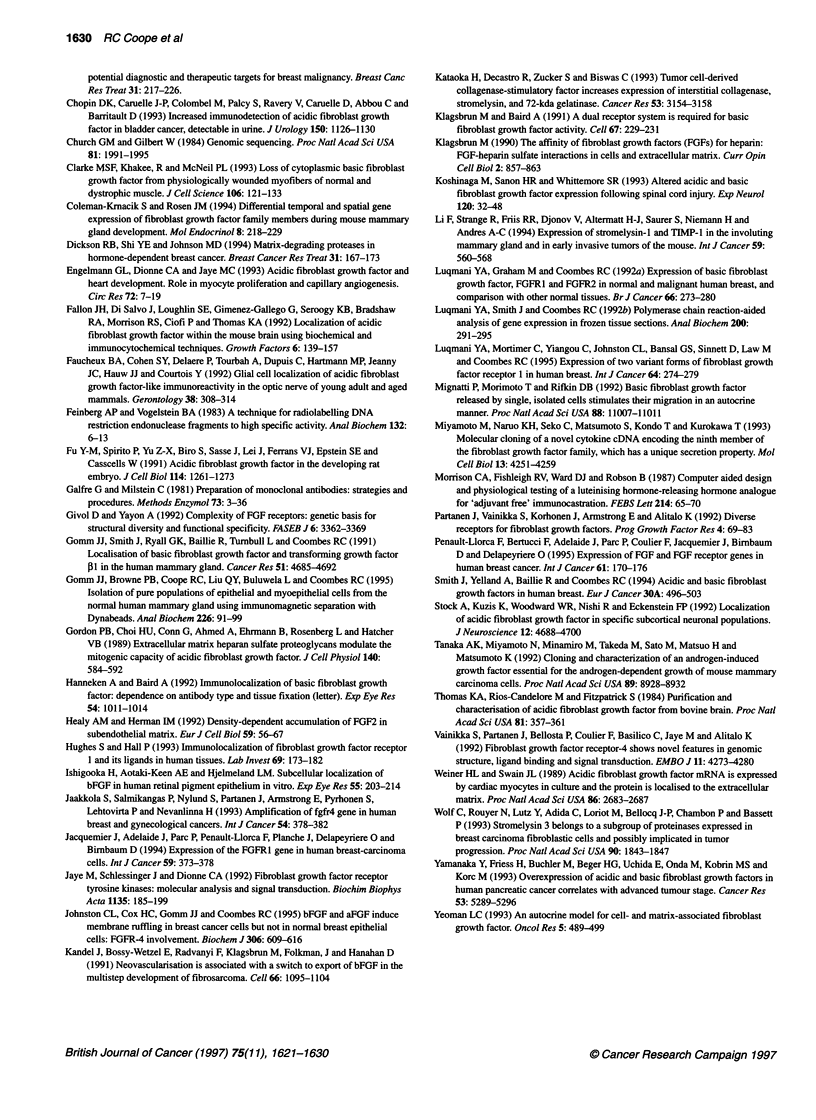# The location of acidic fibroblast growth factor in the breast is dependent on the activity of proteases present in breast cancer tissue.

**DOI:** 10.1038/bjc.1997.277

**Published:** 1997

**Authors:** R. C. Coope, P. J. Browne, C. Yiangou, G. S. Bansal, J. Walters, N. Groome, S. Shousha, C. L. Johnston, R. C. Coombes, J. J. Gomm

**Affiliations:** CRC Department of Medical Oncology, Charing Cross and Westminster Medical School, London, UK.

## Abstract

**Images:**


British Journal of Cancer (1997) 75(11), 1621–1630
© 1997 Cancer Research Campaign

### The location of acidic fibroblast growth factor in the breast is dependent on the activity of proteases present in breast cancer tissue

**RC Coope[1], PJ Browne[1], C Yiangou[1], GS Bansal[1], J Walters[2], N Groome[2], S Shousha[3], CL Johnston[1], RC Coombes[1] and JJ Gomm[1]**

[1]CRC Department of Medical Oncology, Charing Cross and Westminster Medical School, St Dunstan's Road, London W6 8RP, UK; [2]School of Biological and Molecular Sciences, Oxford Brookes University, Gipsy Lane Campus, Headington, Oxford OX3 0BP, UK; [3]Department of Histopathology, Charing Cross and Westminster Medical School, London, UK

**Summary** Acidic fibroblast growth factor (FGF1) and two of its receptors, FGFR1 and FGFR4, were localized in cryostat sections of normal, benign and malignant human breast tissue by immunohistochemistry. Without pretreatment, FGF1 staining was mainly seen in normal epithelial cells. However, polymerase chain reaction (PCR) analysis and immunoblotting of isolated normal epithelial and myoepithelial cells showed FGF1 mRNA and protein to be present in both cell types. Following incubation of frozen sections at 37°C in phosphate-buffered saline, FGF1 staining was also revealed in myoepithelial cells and basement membrane adjacent to carcinoma cells. Treatment with protease inhibitors demonstrated that this effect was due to the activity of an endogenous protease. In contrast, FGF1 staining was found to be associated with the stroma adjacent to malignant cells only in the presence of protease inhibitors. FGFR1 and FGFR4 immunostaining was localized to both normal and malignant epithelial cells and to a lesser extent to myoepithelial cells. There was no difference in the staining intensity for the FGF receptors between normal and cancer samples. The change in location of FGF1 between normal and malignant tissues and the sensitivity of stored FGF1 to the action of endogenous proteases raises the possibility of both autocrine and paracrine roles for FGF1 in the normal and malignant human breast.

**Keywords:** breast cancer; fibroblast growth factor 1; protease; immunohistochemistry

Fibroblast growth factor 1 (FGF1) belongs to a family of multifunctional polypeptides that are involved in a wide array of biological processes, which include cellular proliferation and differentiation, angiogenesis, chemotaxis, embryonal development and tissue repair (Basilico and Moscatelli, 1992). To date, the FGFs consist of a family of nine homologous polypeptide growth factors that include FGF1 (acidic FGF), FGF2 (basic FGF), FGF3 (*int*-2), FGF4 (*hst*-1/Kaposi FGF), FGF5, FGF6 (*hst*-2), FGF7 (keratinocyte growth factor), FGF8 (androgen-induced growth factor) and FGF9 (glial-activating factor) (Basilico and Moscatelli, 1992; Tanaka et al, 1992; Miyamoto et al, 1993). These proteins share 35–50% overall homology of their amino acid sequences (Basilico and Moscatelli, 1992; Givol and Yayon, 1992).

Unlike other members of the family, FGF1, FGF2 and FGF9 are synthesized without a signal peptide sequence and thus may remain sequestered in the cell (Basilico and Moscatelli, 1992; Cao and Pettersson, 1993). However, release of FGF may occur through leakage from damaged cells or from viable cells via a novel mechanism (Mignatti et al, 1992; Cao and Pettersson, 1993). Yeoman (1993) has postulated that proteoglycan-bound FGF may be released from the cell surface or extracellular matrix by the action of proteases, and Briozzo et al (1991) have shown that MCF7 breast cancer cells secrete cathepsin D, which is able to digest the extracellular matrix and release stored FGF2, which was then internalized by the MCF7 cells.

*Received 10 June 1996*
*Revised 26 November 1996*
*Accepted 3 December 1996*

*Correspondence to:* J Gomm

As FGF1 was first purified from brain tissue (Thomas et al, 1984) it is not surprising that its localization was primarily identified in neural tissue such as brain (Fallon et al, 1992; Stock et al, 1992), spinal cord (Koshinaga et al, 1993), optic nerve (Faucheux et al, 1992) and the eye (Caruelle et al, 1989). Hughes and Hall (1993) conducted a large immunohistochemical study of normal human adult tissues using a polyclonal antibody to FGF1. Their results show intense staining for FGF1 in the heart, glomerula of the kidney, urothelium and placenta and moderate immunoreactivity in a wide range of tissues, including breast glandular epithelium. In the normal virgin mouse mammary gland, high levels of FGF1 mRNA were found in partially purified breast organoids and had identical expression to cytokeratin 18, a specific marker for epithelial cells. Normal stroma contained little FGF1 mRNA and there was a lower level of expression in tumorigenesis (Coleman-Krnacik and Rosen, 1994). We have previously shown that FGF1 and FGF2 are both present in human breast tissue (Gomm et al, 1991; Luqmani et al, 1992*a*; Smith et al, 1994) and that they are both mitogenic for breast cancer cell lines (Smith et al, 1994; Johnston et al, 1995). Our studies measuring FGF1 mRNA by reverse transcriptase polymerase chain reaction (RT-PCR) and protein levels by Western blotting of breast tissue samples suggested a reduction in FGF1 synthesis in breast cancer compared with normal breast tissue (Bansal et al, 1995). Similar mRNA results have also been demonstrated using Northern blotting by

Anandappa et al (1994). Using immunostaining techniques, we have localized FGF2 in the myoepithelial cells in paraffin sections of normal breast, but we did not find it in normal or malignant epithelial cells (Gomm et al, 1991), and in a preliminary study we demonstrated staining for FGF1 in normal but not malignant breast epithelial cells (Bansal et al, 1995). We have also detected FGF1 in the conditioned medium from breast tumour biopsies, using a bioassay system, but we were unable to specify whether the signal was produced by tumour cells or by a stromal element (Smith et al, 1994). This breast cancer conditioned-medium was also found to be mitogenic for breast cancer cell lines (Smith et al, 1994).

The response of cells to extracellular FGFs is thought to be mediated through the formation of a ternary complex of FGF, heparan sulphate proteoglycan and high-affinity plasma membrane receptor (Klagsbrun and Baird, 1991; Givol and Yayon, 1992). The high-affinity receptors for FGF belong to the tyrosine kinase superfamily of receptors. So far, receptors encoded by at least four separate genes (FGFR1–4) have been identified (Basilico and Moscatelli, 1992; Givol and Yayon, 1992; Jaye et al, 1992; Partanen et al, 1992). This family of receptors is further complicated by an array of spliced variants that vary in their extra- or intracellular domains, resulting in potentially truncated forms (Givol and Yayon, 1992; Jaye et al, 1992; Partanen et al, 1992). The function of most of these receptor isoforms is unknown. We have demonstrated the presence of FGFR1 and FGFR2 mRNA in both normal and breast cancer samples as well as a panel of breast cell lines and normal human tissues (Luqmani et al, 1992*a*), and Jacquemier et al (1994) have also demonstrated the presence of FGFR1 mRNA in normal and malignant breast epithelial cells using in situ hybridization. Since then we have shown that breast cancer tissues and cell lines express a preponderance of mRNA and protein for the two-loop variant form of FGFR1 as opposed to the full-length three-loop form (Luqmani et al, 1995). Using immunohistochemistry, Hughes and Hall (1993) have described intense positivity for FGFR1 in breast tissues, which was found to be localized within myoepithelial cells of normal breast samples. To our knowledge no studies have as yet been published that describe the localization of FGFR1 protein in breast cancer nor FGFR4 protein in the normal or malignant breast.

In order to understand more fully the role of FGF1 in the mammary gland we examined its localization and that of two of its receptors, FGFR1 and FGFR4 (Partanen et al, 1992), in a variety of human breast tissues. Immunostaining was carried out on cryostat sections of 78 samples using our own specific monoclonal antisera against FGF1 and FGFR1 and a commercial antibody against FGFR4. To identify further the sites of FGF1 storage and synthesis Western blotting and RT-PCR analyses were carried out on purified populations of normal breast epithelial and myoepithelial cells. In addition, we describe here a novel method for assessing the activity of endogenous proteases on FGF1 distribution in frozen breast tissue.

### MATERIALS AND METHODS

### Antibodies

A mouse monoclonal antibody was raised against a synthetic peptide, corresponding to amino acids 60–98 of the FGF1 molecule. This sequence represents part of the FGF1 molecule that has the least homology with FGF2. The antigen used to raise the FGFR1 antibody was in an area of the molecule judged by computer analysis to have high antigenicity and consisted of amino acids 816–822 at the C-terminus of FGFR1. This antibody will detect both alpha and beta forms of the FGFR1 receptor. In the case of FGFR4, the rabbit polyclonal antibody raised to amino acids 789–802 was purchased from Santa Cruz Biotechnology.

Briefly, the FGF1 and FGFR1 peptides were synthesized using the Fmoc method (Atherton and Sheppard, 1985). The peptides were prepared using a NovaSyn Crystal automated peptide synthesizer on a KA (Kieselguhr/polydimethyl/acrylamide) resin (Calbiochem Novabiochem) and peptide purity was checked by reverse-phase high-performance liquid chromatography (HPLC). The peptides were coupled to a purified protein derivative of tuberculin (PPD) (Morrison et al, 1987) and were then injected into female Balb/c mice. The spleen, from a selected mouse, was removed and the splenocytes fused with Sp2/O myeloma cells as previously described (Galfre and Milstein, 1981). Hybridoma supernatants were screened by ELISA before selection for recloning, after which the antibodies were isotyped and total IgG concentrations were evaluated.

### Immunohistochemical staining for FGF1

Tissue from a total of 78 breast biopsies (Table 1) were immediately snap-frozen in liquid nitrogen and embedded in OCT (Optimum cutting tissue) (Raymond A Lamb, London, UK). Tissue sections (8–10 μm) were cut and mounted on Vectabond-coated slides (Vector Labs, Peterborough, UK). Immunostaining was performed using an indirect peroxidase technique. Briefly, frozen sections were fixed in 3.7% formaldehyde in phosphate-buffered saline (PBS) for 10 min and then treated with ice-cold acetone, 50% followed by 100%, for 5 min each. Sections were then washed in PBS (pH 7.2) before blocking with normal goat serum (Vector) [10% in PBS with 5% bovine serum albumin (BSA)] for 30 min at room temperature. This preincubation buffer was then discarded and, without washing, sections were incubated in the mouse monoclonal antibody to FGF1 (3 $\mu g\ ml^{-1}$) or non-immune mouse IgG at the same concentration, diluted in blocking buffer. After 90 min incubation at room temperature in a moist chamber, sections were washed in PBS and incubated in anti-mouse IgG peroxidase conjugate (Sigma Chemical, Poole, Dorset, UK), at a dilution of 1:250 in PBS containing 5% BSA and 10% normal human serum (Sigma). Sections were incubated for a furthur 90 min before washing in PBS and the substrate was developed in a 0.05% solution of 3,3′-diaminobenzidine (Sigma). Sections were counterstained in Gill's haematoxylin. To test the

**Table 1** Histological diagnosis of breast tissue biopsies used in immunostaining for FGF1, FGFR1 and FGFR4

| Histological diagnosis | FGF1 | FGFR1/FGFR4 |
|---|---|---|
| Normal | 23 | 18 |
| Fibroadenoma | 9 | 6 |
| Fibrocystic change | 2 | 1 |
| Lactating breast | 1 | 1 |
| Invasive ductal carcinoma | 30 | 16 |
| Invasive lobular carcinoma | 3 | 3 |
| Ductal carcinoma *in situ* | 9 | 7 |
| Mucinous carcinoma | 1 | 0 |
| Total numbers | 78 | 52 |

 © Cancer Research Campaign 1997

specificity of the anti-FGF1 antibody, dilute antiserum, at a concentration of 3 μg ml$^{-1}$, was incubated overnight at 4°C with an excess (300 μg ml$^{-1}$) of the immunizing peptide, prior to immunostaining.

### Immunohistochemical staining for FGFR1 and FGFR4

A three-stage avidin–biotin complex (ABC) immunoperoxidase technique was used for the localization of FGFR1 and FGFR4. Frozen sections were fixed in 3.7% formaldehyde in PBS for 10 min and permeabilized in ice-cold acetone, 50% followed by 100%, for 5 min each. Sections were then washed in PBS and blocked for endogenous biotin following the protocol included with a biotin blocking kit (Vector). After further washes in PBS, sections were preincubated with normal goat serum (in the case of FGFR4) or normal horse serum (in the case of FGFR1) (10% in PBS with 5% BSA) for 30 min at room temperature before incubation with the primary antibody (0.5 μg ml$^{-1}$) or non-immune mouse or rabbit IgG at equivalent concentrations, overnight at 4°C. The following day sections were washed and incubated in biotinylated second antibodies (1:200, diluted in PBS with 10% human serum) for 30 min and finally incubated in Vectastain ABC reagent (Vector) for 1 h at room temperature. Staining was visualized using 0.05% 3,3′-diaminobenzidine and counterstained with Gill's haematoxylin.

### Assay for endogenous proteases

Unfixed frozen sections were incubated in either PBS only at pH 7.2 or the same buffer containing a mixture of the serine protease inhibitors 6-aminohexanoic acid (100 mM), benzamidine hydrochloride (5 mM) and phenylmethylsulphonylfluoride (PMSF) (1 mM), the thiol protease inhibitor *N*-ethylmaleimide (1 mM) and the metalloproteinase inhibitor disodium EDTA (10 mM) for 2 h at 37°C. Sections were then washed in three changes of PBS and stained for FGF1 by the indirect peroxidase technique described above.

**Figure 1** Western immunoblots for FGF1. (**A**) Showing the specificity of the anti-FGF1 monoclonal antibody for FGF1. Lane 1, 15 ng of recombinant FGF1; lane 2, 15 ng of recombinant FGF2. In lanes 3 and 4, 15 ng of FGF1 and FGF2 respectively were incubated with the monoclonal anti-FGF1 antibody, which had been preincubated with an excess of the immunizing peptide. (**B**) Separated normal breast cell fractions incubated with anti-FGF1 antibody. E, epithelial cells; M, myoepithelial cells

### RNA extraction, reverse transcription and PCR amplification

Breast organoids were prepared from reduction mammoplasty tissue as described previously (Gomm et al, 1995). From organoid preparations we were then able to obtain purified populations of epithelial and myoepithelial cells by immunomagnetic separation (Gomm et al, 1995). PCR and immunostaining for the epithelial and myoepithelial markers epithelial membrane antigen (EMA), common acute lymphoblastic leukaemia antigen (CALLA) and cytokeratins 18, 19 and 14 have shown these separated cell populations to be consistently 97–99% pure (Gomm et al, 1995).

mRNA from pure cell populations of epithelial and myoepithelial cells were extracted using the Dynabeads mRNA Direct kit (Dynal, UK). For reverse transcription (RT), first-strand synthesis was carried out using Moloney murine leukaemia virus (MMLV) reverse transcriptase and 2 μg of RNA in a volume of 20 μl. An aliquot (1 μl) of RT product was added to 99 μl of the PCR mixture containing 1 unit of *Taq* polymerase, 200 ng each of the actin primers 5′-CATCTCTTGCTCGAAGAAGTCCA-3′ and 5′-ATCATGTTTGAGACCTTCAA-3′ plus 200 ng each of either the FGF1 primers, 5′-GATGGCACAGTGGATGGGAC-3′ and 5′-AAGCCCGTCGGTGTCCATGG-3′ or the FGFR1, 5′-CCTCCTCTTCTGGGCTGTGCT-3′ and 5′-TCTTTTCTGGGGATGTCC-3′ or FGFR4 primers, 5′-GGTCCTGCTGAGTGTGCCTG-3′ and 5′-GGGGTAACTGTGCCTATTCG-3′.

PCR products were chloroform extracted and 10 μl of each sample was electrophoresed on 1% agarose gels and alkali blotted overnight, for subsequent hybridization. The FGF1 and actin samples were hybridized to cDNA randomly labelled with [α-$^{32}$P]dCTP, using the random primer method (Feinberg and Vogelstein, 1983) and the FGFR1 and FGFR4 samples were hybridized with internal oligonucleotides that were end-labelled with [γ-$^{32}$P]ATP. Hybridization was carried out as described by Church and Gilbert (1984). The hybridized filters were analysed by phosphoimaging. The values for FGF1, FGFR1 and FGFR4 were normalized by dividing the signals by that for actin.

### Immunoblotting

The specificity of the FGF1 antibody used in immunohistochemistry was determined by sodium dodecyl sulphate polyacrylamide gel electrophoresis (SDS-PAGE) followed by Western blotting.

© Cancer Research Campaign 1997

**Table 2** Expression of FGF1, FGFR1 and FGFR4 mRNA in separated breast cell populations

| | Epithelial cells | Myoepithelial cells |
|---|---|---|
| FGF1[a] | +++ | +++ |
| FGFR1[a] | ++ | ++ |
| FGFR4[a] | ++ | ++ |

[a]Results normalized to actin.

FGF1 and FGF2 peptides were mixed with SDS-PAGE sample buffer and electrophoresed on a 15% polyacrylamide gel. Separated proteins were transferred onto a nitrocellulose membrane overnight at 4°C. Non-specific binding sites were blocked with 3% milk powder in PBS-T (PBS + 0.1% Tween 20) for 1 h at room temperature before incubation of the membrane with either anti-FGF1 antibody or the same antiserum after it had been preincubated with an excess of the FGF1 immunizing peptide. After washing, blots were incubated with an anti-mouse IgG-peroxidase conjugate and then washed five times in PBS-T. Bands were visualized using the ECL method (Amersham). Purified populations of normal breast epithelial and myoepithelial cells were lysed in SDS-PAGE sample buffer and 40 μg of protein electrophoresed, blotted and stained in the same manner as the standards above.

### RESULTS

### Immunoblotting

The anti-FGF1 antibody recognized a protein band at 18 kDa consistent with the molecular weight of recombinant FGF1 and showed no cross-reactivity with FGF2 (Figure 1A). The FGF1 band was absent when the membrane was incubated with anti-FGF1 antibody, which had been preincubated with an excess of the FGF1 peptide used to raise the antibody (Figure 1A). Western hybridization of the separated normal epithelial and myoepithelial cells with the same antibody to FGF1 showed both cell types to have a band at 18 kDa (Figure 1B).

### FGF1, FGFR1 and FGFR4 expression by PCR

PCR conditions were optimized as previously described (Luqmani et al, 1992*a*) to ensure that amplification was in the linear phase. A total of 35 cycles of PCR for the epithelial cell marker EMA and the myoepitheial cell marker CALLA demonstrated the purity of the separated cell populations (Gomm et al, 1995). Eighteen cycles of PCR were selected for the estimation of actin levels and 28 and 40 cycles for FGF1, FGFR1 and FGFR4. All samples produced the expected product size of 135 bp for FGF1, 465 bp for FGFR1 or 402 bp for FGFR4. In each case a single band corresponding to 319 bp was also seen for actin. Table 2 shows that both normal

**Figure 2** Peroxidase–haematoxylin staining of frozen sections of normal human breast. (**A**) Incubated with non-immune mouse IgG (original magnification, ×200). (**B**) Incubation with anti-FGF1 antibody results in intense staining of epithelial cells but not myoepithelial cells (original magnification, ×200). (**C**) Higher power photomicrograph shows FGF1 staining localized to cytoplasm and plasma membrane of normal epithelial cells (original magnification, ×400). (**D**) Incubated with antigen-absorbed anti-FGF1 antibody (original magnification, ×200)

© Cancer Research Campaign 1997

**Figure 3** Peroxidase–haematoxylin staining of frozen sections of malignant breast for FGF1. (**A**) DCIS showing no staining. (**B**) DCIS with scattered positively stained cells. (**C**) Invasive ductal carcinoma with no immunoreactivity. (**D**) Invasive ductal carcinoma with scattered positivity. (**E**) Invasive ductal carcinoma with pale, homogeneous FGF1 staining. (**F**) Invasive lobular carcinoma, with areas of pale FGF1 staining (original magnification of all sections, ×200)

epithelial and myoepithelial cells express mRNA for FGF1, FGFR1 and FGFR4.

### Distribution of FGF1 staining

A total of 47 samples of normal breast were examined. These consisted of adjacent normal tissue taken from around carcinomas ($n$ = 24) and reduction mammoplasty tissue ($n$ = 23). Staining was confined to the epithelial cells in ducts and acini (Figure 2B) and appeared at the light microscopy level to be associated with both the cytoplasm and plasma membrane (Figure 2C). Myoepithelial cells and stroma were essentially unstained. The number of cells staining and the intensity were variable. However, strongest staining appeared to be in the epithelial cells of the main ducts. Positively stained normal epithelial cells were also identified adjacent to neoplastic tissue and a similar pattern of staining was observed. Using tissue sections of normal breast, immunohistochemistry was performed after preincubation of the antibody with a 100-fold excess of the immunizing peptide (Figure 2D). By blocking anti-FGF1 antibody binding with the peptide, all FGF1 immunostaining was completely abolished. Two examples of fibrocystic change and nine fibroadenomas were examined and all showed epithelial cell staining for FGF1. One case of lactating breast also showed scattered positive staining in the ducts; however, all lactating acini were negative for FGF1.

Nine cases of ductal carcinoma in situ (DCIS) were examined. Three cases of solid DCIS and three cases of pure comedo carcinoma

© Cancer Research Campaign 1997

**Figure 4** Peroxidase–haematoxylin staining of unfixed frozen sections of invasive ductal carcinoma of the breast for FGF1. Adjacent normal duct (**A** and **B**) and invasive cancer (**C–F**). **A**, **C** and **E**: Preincubation of sections with PBS for 2 h at 37° C. **B**, **D** and **F**: Preincubation of sections with PBS plus a cocktail of protease inhibitors. (**A**) PBS treatment causes revealing of FGF1 epitope in myoepithelial cells of adjacent normal duct. (**B**) Inclusion of protease inhibitors prevents FGF1 epitope on myoepithelial cells being accessible to anti-FGF1 antibody. (**C**) PBS treatment also results in FGF1 staining in the basement membrane surrounding tumour islands. (**D**) With the addition of protease inhibitors no basement membrane FGF1 staining is seen. (**E**) After incubation in PBS only, stroma adjacent to malignant epithelial cells is negative for FGF1. (**F**) Treatment with protease inhibitors prevents release of stromal FGF1 and thus stabilizes FGF1 staining (original magnification of all sections, ×200)

were entirely negative (Figure 3A). Two cases of combined solid and comedo DCIS and one micropapillary case had some scattered positive cells (Figure 3B). Out of a total of 30 cases of invasive ductal carcinoma only one case showed pale but homogeneous staining of neoplastic epithelial cells (Figure 3E) and three others showed areas of scattered positivity (Figure 3D). The remainder were entirely negative (Figure 3C). Of three cases of invasive lobular carcinoma, all cases showed some areas of pale staining (Figure 3F). One case of mucinous carcinoma was negative.

In view of previous studies showing a redistribution of FGF2 staining, dependent on the type of tissue fixation used (Hanneken and Baird, 1992; Healy and Herman 1992; Ishigooka et al, 1992) and because of our own experience in immunostaining for this growth factor (results not shown), we examined the effects of different fixation methods on FGF1 localization. Cryostat sections of both normal and malignant breast tissues fixed either in acetone (50% followed by 100%) or 3.7% formaldehyde in PBS alone, or formaldehyde followed by acetone gave the same

© Cancer Research Campaign 1997

pattern of FGF1 staining whichever method was used (results not shown).

To determine whether the distribution of FGF1 in the breast was affected by the action of any endogenous proteases present in either normal or malignant breast tissues we examined the effect of incubating unfixed frozen sections at 37°C for 2 h in PBS, with and without a cocktail of protease inhibitors (see method) on the pattern of FGF1 staining. We found that treatment with PBS at pH 7.2 alone resulted in the appearance of FGF1 staining in the myoepithelial cells of normal ducts adjacent to malignant tissue and this was sometimes accompanied by a loss of epithelial cell staining (Figure 4A). This effect was not seen in normal ducts in reduction mammoplasty specimens (Table 3). Residual myoepithelial cells and basement membrane surrounding islands of malignant epithelial cells were also positive for FGF1 following incubation in PBS at 37°C (Figure 4C).

When protease inhibitors were included in the PBS buffer the myoepithelial cell and basement membrane staining was lost and again FGF1 staining was only seen in normal epithelial cells (Figure 4B and D). Thus, the presence of protease inhibitors

**Figure 5** Peroxidase–haematoxylin staining of frozen sections for FGFR1 and FGFR4. **A**, **C** and **E**: Normal breast. **B**, **D** and **F**: Invasive ductal carcinoma of the breast. (**A**) Section of normal breast incubated with non-immune mouse IgG. (**B**) Section of malignant breast incubated with non-immune rabbit IgG. (**C** and **D**) Sections incubated with anti-FGFR1 antibody show equivalent cytoplasmic staining of normal and malignant epithelial cells; myoepithelial cells exhibit paler staining. (**E** and **F**) Sections incubated with anti-FGFR4 antibody show similar but paler staining distribution to anti-FGFR1 (original magnification of all sections, ×200).

**Table 3** Comparison of the effects of PBS and protease inhibitor treatments on FGF1 immunostaining in normal vs adjacent normal breast ducts

| Treatment | Epithelial cells | Myoepithelial cells | Basement membrane | Stroma |
|---|---|---|---|---|
| Normal duct | | | | |
| None | + | – | – | – |
| PBS | + | – | – | – |
| Protease inhibitors | + | – | – | – |
| Adjacent normal duct | | | | |
| None | + | – | – | – |
| PBS | +/– | + | + | – |
| Protease inhibitors | + | – | – | + |

prevented the FGF1 epitopes being accessible to the antibody on the myoepithelial cells and the basement membrane, suggestive of the presence of an endogenous protease associated with the 'normal' ducts adjacent to cancer tissue, which is active at a neutral pH. An additional and significant effect of the incubation of unfixed sections in PBS at 37°C in the presence of protease inhibitors was the revelation of FGF1 staining in the stroma of all the breast cancer samples treated, particularly in association with malignant cells (Figure 4F). In no case did treatment with PBS or protease inhibitors reveal additional FGF1 staining in the malignant epithelial cells (Figure 4C–F) or in normal stroma. Incubation of the anti-FGF1 antibody with immunizing peptide caused a loss of staining in all cases.

### Immunohistochemistry for FGFR1 and FGFR4

All normal and benign breast tissue samples showed cytoplasmic staining for FGFR1 in the epithelial cells (Figure 5C). Myoepithelial cells were also stained but less strongly. No staining of stromal cells was seen. This correlates with our previous PCR results, which showed FGFR1 mRNA in microdissected keratin 19-expressing cells but not in breast stroma (Luqmani et al, 1992*b*). FGFR1 immunoreactivity was also found in the ductal and acinar epithelial cells of lactating breast tissue. Sections of 16 invasive ductal and three invasive lobular carcinomas showed homogeneous cytoplasmic staining of epithelial cells in all sections (Figure 5D). Seven cases of DCIS were also positive, as was the adjacent normal breast tissue present in 11 cases. The pattern of FGFR4 immunoreactivity showed an identical distribution to that seen for FGFR1, but staining was not quite as intense (Figure 5E and F). There appeared to be no difference in staining intensity between normal and malignant epithelial cells for both FGF receptors.

### DISCUSSION

This study represents the first detailed account of the localization of FGF1 and two of its receptors in breast tissue. Our results show that the distribution of FGF1 is different between normal breast and breast carcinoma: in the former it is present in the luminal epithelial and myoepithelial cells, whereas in the latter it is also seen in the basement membrane and stromal tissue surrounding the malignant epithelial cells, which are essentially negative for FGF1. Our findings also demonstrate the presence of receptors for FGF1 in both normal and malignant epithelial cells, suggesting important autocrine and paracrine roles for this growth factor in both the normal and neoplastic breast.

The pattern that emerges of FGF1 localization in the breast is a complex one but does give us some clues as to how the function of this growth factor may be modified in the transition from the normal to neoplastic state. Before incubation of unfixed frozen sections in PBS at 37°C, with or without protease inhibitors, although we were always able to demonstrate FGF1 staining in normal epithelial cells, we were unable to see FGF1 staining in the myoepithelial cells, basement membrane and stromal tissue surrounding carcinoma cells. As we have shown here, the differences seen in the distribution of FGF1 staining are not due to the fixation method used nor could they have been due to artefacts of freezing or the section cutting process (Clarke et al, 1993) as there were major and specific changes in the FGF1 staining pattern dependent on whether unfixed sections were treated with PBS alone or PBS plus protease inhibitors, and especially between cancer and normal breast tissues. It is more likely that these results are because of the presence of at least one endogenous protease in malignant breast tissue, which is either absent or inactive in the normal breast, as similar treatment caused no change in the staining pattern in normal tissues.

The question now arises as to the nature and role of the proteases involved in the sequestration and function of FGF1 in breast cancer. The revealing of the FGF1 epitope in the myoepithelial cells in adjacent normal ducts but not normal ducts in reduction mammoplasty tissue suggests a transition from a normal to a preneoplastic state that involves the synthesis or activation of a protease. The metalloprotease stromelysin-1 has been detected in myoepithelial cells surrounding preneoplastic lesions (Li et al, 1994) and matrilysin mRNA is expressed in both neoplastic breast epithelial cells and non-neoplastic epithelial cells associated with breast cancer (Wolf et al, 1993).

The stromal FGF1 staining seen only in association with malignant areas following incubation with protease inhibitors was present in all the cancers studied. As this staining was lost when sections were incubated in PBS at 37°C this was indicative of the effect of a protease present in the stroma that acts to release FGF1 from its storage sites. This may be a different protease to the one acting on the ducts themselves. Many proteases, including the collagenases, gelatinases, stromelysins, cathepsins and urokinase-type plasminogen activator are thought to have roles in cell-surface proteolysis and invasion in breast cancer (Chen et al, 1994; Dickson et al, 1994), and more specific combinations of protease inhibitors will be required to identify the nature of the enzymes involved in the FGF1 transition seen here. The presence of FGF1 in the stroma of cancer tissues also now explains the source of the released FGF1 that we detected by bioassay of conditioned medium from breast tumour biopsies (Smith et al, 1994). Stromal FGF1 may also be the ligand for the FGF receptors expressed on malignant epithelial cells, perhaps being released from storage by the action of a secreted protease from the cancer cells themselves (Briozzo et al, 1991). We have shown that both FGF1 and conditioned medium from breast cancer biopsies is mitogenic for MCF7 and T47D breast cancer cell lines (Smith et al, 1994; Johnston et al, 1995). Alternatively, cancer cells may stimulate the surrounding fibroblasts to produce their own matrix proteases. Stromal cells surrounding invasive breast carcinoma cells have been shown to synthesize the matrix metalloproteinase, stromelysin-3 (Basset et al, 1991; Wolf et al, 1993), and lung carcinoma cells release a factor that increases collagenase expression in fibroblast cells (Kataoka et al, 1993).

© Cancer Research Campaign 1997

Our immunostaining results agree with work by Hughes and Hall (1993), in which FGF1 was found to be localized to the glandular epithelium of normal breast. But the pattern of FGF1 distribution in breast cancer seems to differ from that seen in other tissues such as pancreas, brain and bladder where FGF1 was found to be overexpressed in malignant tissue (Akutsu et al, 1991; Yamanaka et al, 1993; Chopin et al, 1993). However, the mechanism of FGF1 action in breast cancer may be different from these tissues. Although our immunostaining results show a loss of FGF1 staining in the transition from normal to malignant epithelial cells, this is replaced by intense stromal FGF1 staining closely associated with cancer cells which is sensitive to protease release.

As our previous results (Bansal et al, 1995) have suggested a reduction in FGF1 synthesis in breast cancer compared with normal breast tissue, this may mean that the FGF1 stromal staining that we see in breast cancer sections may represent stored and not newly synthesized FGF1. Similar to our immunostaining results using tissue sections, we have found breast cancer cell lines to be negative for FGF1 protein (Bansal et al, 1995), but it is possible that FGF1 is released into the stroma at an early stage in the transition to malignancy. The alterations in the FGF1 staining pattern that we see in the 'normal' ducts adjacent to cancer cells following PBS incubation may be representative of this change in FGF1 storage. Interestingly, Kandel et al (1991) found that there is a switch from intracellular to extracellular FGF when normal fibroblasts undergo a progression to aggressive fibromatosis and fibrosarcoma in transgenic mice.

The immunostaining of normal epithelial cells appears to be both cytoplasmic and membrane associated. Thus the activity of breast proteases may be twofold, firstly releasing FGF1 from its site on the epithelial cell membrane when it becomes sequestered by extracellular heparan sulphate proteoglycans (HSPGs) in the stroma, then at a later stage the FGF1/HSPG complex could be released from these storage sites allowing it to act on the external high-affinity receptors that we have demonstrated to be present on malignant cells. Such a specialized autocrine model for FGF action has been proposed by Yeoman (1993). Both FGF1 and FGF2 have been found bound to HSPGs either on the cell surface or in the extracellular matrix (Klagsbrun, 1990) and HSPG-bound FGF1 has been shown to be 100× more mitogenic than heparin-bound FGF1 (Gordon et al, 1989). FGF1 has been localized extracellularly in vivo in the developing heart (Engelmann et al, 1993), tooth (Cam et al, 1992), lungs, digestive system, CNS and eye (Fu et al, 1991) where it is thought to act both as a paracrine growth factor as well as stimulating capillary and neural invasion. Weiner and Swain (1989) have shown that neonatal cardiac myocytes deposit FGF1 into their extracellular matrix and FGFR1 transcipts have been localized on developing cardiomyocytes (Engelman et al, 1993). Thus, tumorogenesis may mimic development in its mechanism of FGF1 action.

FGF1 binds to FGFR1 and FGFR4 with similar high affinities but FGFR4 has been shown to have a far stronger affinity for FGF1 than any other member of the FGF family (Vainikka et al, 1992). About 10% of breast cancers have been shown to contain amplified levels of the genes for FGFR1 (Adnane et al, 1991; Jacquemier et al, 1994) and FGFR4 (Jaakkola et al, 1993). Jacquemier et al (1994) also reported overexpression of FGFR1 mRNA in 14.5% of breast tumours and Penault-Llorca et al (1995) found both FGFR1 and FGFR4 mRNA to be expressed at high levels in 22% and 32% of breast cancers respectively. Our immunostaining results, however, revealed homogeneous staining for FGFR1 and FGFR4 protein in all breast epithelial cells, with no apparent difference in staining intensity between normal and malignant tissues. This agrees with our results using PCR to detect FGFR mRNA in breast tissue extracts (Luqmani et al, 1992*a*; 1995). To our knowledge this is the first incidence of a growth factor receptor that is present at equivalent levels in normal and cancer tissue.

In conclusion, using immunohistochemistry for FGF1 and two of its receptors, together with a novel in situ assay for endogenous proteases we have shown that the role of this growth factor in the breast may depend on its location. It is possible that in the normal gland FGF1 may be largely sequestered on epithelial cells and unable to interact significantly with cell-surface receptors but once released into the extracellular matrix by the inappropriate expression of proteases in breast cancer it becomes more bioavailable to the epithelial cells.

### ACKNOWLEDGEMENTS

This work was supported by a grant from The Cancer Research Campaign. C Yiangou was funded by the Buckle Family Trust. The authors wish to thank Dr Y Cao at the Ludwig Institute for Cancer Research, Stockholm, Sweden, and Dr F Bertolero at Farmitalia, Milan, Italy, for kindly providing us with the FGF1 and FGF2 standards used in immunoblotting. We would also like to thank Mrs Jean Sterling and Miss Mandy Lee for their help in preparing this manuscript.

### REFERENCES

Adnane J, Gaudray P, Dionne CA, Crumley G, Jaye M, Schlessinger J, Jeanteur P, Birnbaum D and Theillet C (1991) BEK and *FLG*, two receptor members of the FGF family, are amplified in subsets of human breast cancers. *Oncogene*, **6**: 659–663

Akutsu Y, Aida T, Nakazawa S and Asano G (1991) Localisation of acidic and basic fibroblast growth factor mRNA in human brain tumours. *Jpn J Cancer Res* **82**: 1022–1027

Anandappa SY, Winstanley JHR, Leinster S, Green B, Rudland PS and Barraclough R (1994) Comparative expression of fibroblast growth factor mRNAs in benign and malignant breast disease. *Br J Cancer* **69**: 772–776

Atherton E and Sheppard RC (1985) Solid phase peptide synthesis using N-fluorenylmethoxycarbonyl amino acid penta-fluorophenyl esters. *J Chem Soc Chem Commun* 165–166

Bansal GS, Johnston CL, Coope RC, Gomm JJ, Luqmani YA, Coombes RC and Yiangou C (1995) Expression of fibroblast growth factor 1 is lower in breast cancer than in normal human breast. *Br J Cancer* **72**: 1420–1426

Basilico C and Moscatelli D (1992) The FGF family of growth factors and oncogenes. *Adv Cancer Res* **5**: 115–164

Basset P, Bellocq JP, Wolf C, Stoll I, Hutin P, Limacher JM, Podhajcer OL, Chenard MP, Rio MC and Chambon P (1991) A novel metalloproteinase gene specifically expressed in stromal cells of breast carcinomas. *Nature* **348**: 699–704

Briozzo P, Badet J, Capony F, Pieri I, Montcourrier P, Barritault D and Rochefort H (1991) MCF7 mammary cancer cells respond to bFGF and internalize it following its release from extracellular matrix: a permissive role of cathepsin D. *Exp Cell Res* **194**: 252–259

Cam Y, Neumann M-R, Oliver L, Raulais D, Janet T and Ruch J-V (1992) Immunolocalization of acidic and basic fibroblast growth factors during mouse odontogenesis. *Int J Dev Biol* **36**: 381–389

Cao Y and Pettersson R (1993) Release and subcellular localization of acidic fibroblast growth factor expressed to high levels in HeLa cells. *Growth Factors* **8**: 277–291

Caruelle D, Groux-Muscatelli B, Gaudric A, Sestier C, Coscas G, Caruelle J-P and Barritault D (1989) Immunological study of acidic fibroblast growth factor (aFGF) distribution in the eye. *J Cell Biochem* **39**: 117–128

Chen W-T, Lee C-C, Goldstein L, Bernier S, Liu CHL, Lin C-Y, Yeh Y, Monsky WL, Kelly T, Dai M, Zhou J-Y and Mueller SC (1994) Membrane proteases as

© Cancer Research Campaign 1997 

potential diagnostic and therapeutic targets for breast malignancy. *Breast Canc Res Treat* **31**: 217–226.

Chopin DK, Caruelle J-P, Colombel M, Palcy S, Ravery V, Caruelle D, Abbou C and Barritault D (1993) Increased immunodetection of acidic fibroblast growth factor in bladder cancer, detectable in urine. *J Urology* **150**: 1126–1130

Church GM and Gilbert W (1984) Genomic sequencing. *Proc Natl Acad Sci USA* **81**: 1991–1995

Clarke MSF, Khakee, R and McNeil PL (1993) Loss of cytoplasmic basic fibroblast growth factor from physiologically wounded myofibers of normal and dystrophic muscle. *J Cell Science* **106**: 121–133

Coleman-Krnacik S and Rosen JM (1994) Differential temporal and spatial gene expression of fibroblast growth factor family members during mouse mammary gland development. *Mol Endocrinol* **8**: 218–229

Dickson RB, Shi YE and Johnson MD (1994) Matrix-degrading proteases in hormone-dependent breast cancer. *Breast Cancer Res Treat* **31**: 167–173

Engelmann GL, Dionne CA and Jaye MC (1993) Acidic fibroblast growth factor and heart development. Role in myocyte proliferation and capillary angiogenesis. *Circ Res* **72**: 7–19

Fallon JH, Di Salvo J, Loughlin SE, Gimenez-Gallego G, Seroogy KB, Bradshaw RA, Morrison RS, Ciofi P and Thomas KA (1992) Localization of acidic fibroblast growth factor within the mouse brain using biochemical and immunocytochemical techniques. *Growth Factors* **6**: 139–157

Faucheux BA, Cohen SY, Delaere P, Tourbah A, Dupuis C, Hartmann MP, Jeanny JC, Hauw JJ and Courtois Y (1992) Glial cell localization of acidic fibroblast growth factor-like immunoreactivity in the optic nerve of young adult and aged mammals. *Gerontology* **38**: 308–314

Feinberg AP and Vogelstein BA (1983) A technique for radiolabelling DNA restriction endonuclease fragments to high specific activity. *Anal Biochem* **132**: 6–13

Fu Y-M, Spirito P, Yu Z-X, Biro S, Sasse J, Lei J, Ferrans VJ, Epstein SE and Casscells W (1991) Acidic fibroblast growth factor in the developing rat embryo. *J Cell Biol* **114**: 1261–1273

Galfre G and Milstein C (1981) Preparation of monoclonal antibodies: strategies and procedures. *Methods Enzymol* **73**: 3–36

Givol D and Yayon A (1992) Complexity of FGF receptors: genetic basis for structural diversity and functional specificity. *FASEB J* **6**: 3362–3369

Gomm JJ, Smith J, Ryall GK, Baillie R, Turnbull L and Coombes RC (1991) Localisation of basic fibroblast growth factor and transforming growth factor β1 in the human mammary gland. *Cancer Res* **51**: 4685–4692

Gomm JJ, Browne PB, Coope RC, Liu QY, Buluwela L and Coombes RC (1995) Isolation of pure populations of epithelial and myoepithelial cells from the normal human mammary gland using immunomagnetic separation with Dynabeads. *Anal Biochem* **226**: 91–99

Gordon PB, Choi HU, Conn G, Ahmed A, Ehrmann B, Rosenberg L and Hatcher VB (1989) Extracellular matrix heparan sulfate proteoglycans modulate the mitogenic capacity of acidic fibroblast growth factor. *J Cell Physiol* **140**: 584–592

Hanneken A and Baird A (1992) Immunolocalization of basic fibroblast growth factor: dependence on antibody type and tissue fixation (letter). *Exp Eye Res* **54**: 1011–1014

Healy AM and Herman IM (1992) Density-dependent accumulation of FGF2 in subendothelial matrix. *Eur J Cell Biol* **59**: 56–67

Hughes S and Hall P (1993) Immunolocalization of fibroblast growth factor receptor 1 and its ligands in human tissues. *Lab Invest* **69**: 173–182

Ishigooka H, Aotaki-Keen AE and Hjelmeland LM. Subcellular localization of bFGF in human retinal pigment epithelium in vitro. *Exp Eye Res* **55**: 203–214

Jaakkola S, Salmikangas P, Nylund S, Partanen J, Armstrong E, Pyrhonen S, Lehtovirta P and Nevanlinna H (1993) Amplification of fgfr4 gene in human breast and gynecological cancers. *Int J Cancer* **54**: 378–382

Jacquemier J, Adelaide J, Parc P, Penault-Llorca F, Planche J, Delapeyriere O and Birnbaum D (1994) Expression of the FGFR1 gene in human breast-carcinoma cells. *Int J Cancer* **59**: 373–378

Jaye M, Schlessinger J and Dionne CA (1992) Fibroblast growth factor receptor tyrosine kinases: molecular analysis and signal transduction. *Biochim Biophys Acta* **1135**: 185–199

Johnston CL, Cox HC, Gomm JJ and Coombes RC (1995) bFGF and aFGF induce membrane ruffling in breast cancer cells but not in normal breast epithelial cells: FGFR-4 involvement. *Biochem J* **306**: 609–616

Kandel J, Bossy-Wetzel E, Radvanyi F, Klagsbrun M, Folkman, J and Hanahan D (1991) Neovascularisation is associated with a switch to export of bFGF in the multistep development of fibrosarcoma. *Cell* **66**: 1095–1104

Kataoka H, Decastro R, Zucker S and Biswas C (1993) Tumor cell-derived collagenase-stimulatory factor increases expression of interstitial collagenase, stromelysin, and 72-kda gelatinase. *Cancer Res* **53**: 3154–3158

Klagsbrun M and Baird A (1991) A dual receptor system is required for basic fibroblast growth factor activity. *Cell* **67**: 229–231

Klagsbrun M (1990) The affinity of fibroblast growth factors (FGFs) for heparin: FGF-heparin sulfate interactions in cells and extracellular matrix. *Curr Opin Cell Biol* **2**: 857–863

Koshinaga M, Sanon HR and Whittemore SR (1993) Altered acidic and basic fibroblast growth factor expression following spinal cord injury. *Exp Neurol* **120**: 32–48

Li F, Strange R, Friis RR, Djonov V, Altermatt H-J, Saurer S, Niemann H and Andres A-C (1994) Expression of stromelysin-1 and TIMP-1 in the involuting mammary gland and in early invasive tumors of the mouse. *Int J Cancer* **59**: 560–568

Luqmani YA, Graham M and Coombes RC (1992*a*) Expression of basic fibroblast growth factor, FGFR1 and FGFR2 in normal and malignant human breast, and comparison with other normal tissues. *Br J Cancer* **66**: 273–280

Luqmani YA, Smith J and Coombes RC (1992*b*) Polymerase chain reaction-aided analysis of gene expression in frozen tissue sections. *Anal Biochem* **200**: 291–295

Luqmani YA, Mortimer C, Yiangou C, Johnston CL, Bansal GS, Sinnett D, Law M and Coombes RC (1995) Expression of two variant forms of fibroblast growth factor receptor 1 in human breast. *Int J Cancer* **64**: 274–279

Mignatti P, Morimoto T and Rifkin DB (1992) Basic fibroblast growth factor released by single, isolated cells stimulates their migration in an autocrine manner. *Proc Natl Acad Sci USA* **88**: 11007–11011

Miyamoto M, Naruo KH, Seko C, Matsumoto S, Kondo T and Kurokawa T (1993) Molecular cloning of a novel cytokine cDNA encoding the ninth member of the fibroblast growth factor family, which has a unique secretion property. *Mol Cell Biol* **13**: 4251–4259

Morrison CA, Fishleigh RV, Ward DJ and Robson B (1987) Computer aided design and physiological testing of a luteinising hormone-releasing hormone analogue for 'adjuvant free' immunocastration. *FEBS Lett* **214**: 65–70

Partanen J, Vainikka S, Korhonen J, Armstrong E and Alitalo K (1992) Diverse receptors for fibroblast growth factors. *Prog Growth Factor Res* **4**: 69–83

Penault-Llorca F, Bertucci F, Adelaide J, Parc P, Coulier F, Jacquemier J, Birnbaum D and Delapeyriere O (1995) Expression of FGF and FGF receptor genes in human breast cancer. *Int J Cancer* **61**: 170–176

Smith J, Yelland A, Baillie R and Coombes RC (1994) Acidic and basic fibroblast growth factors in human breast. *Eur J Cancer* **30A**: 496–503

Stock A, Kuzis K, Woodward WR, Nishi R and Eckenstein FP (1992) Localization of acidic fibroblast growth factor in specific subcortical neuronal populations. *J Neuroscience* **12**: 4688–4700

Tanaka AK, Miyamoto N, Minamiro M, Takeda M, Sato M, Matsuo H and Matsumoto K (1992) Cloning and characterization of an androgen-induced growth factor essential for the androgen-dependent growth of mouse mammary carcinoma cells. *Proc Natl Acad Sci USA* **89**: 8928–8932

Thomas KA, Rios-Candelore M and Fitzpatrick S (1984) Purification and characterisation of acidic fibroblast growth factor from bovine brain. *Proc Natl Acad Sci USA* **81**: 357–361

Vainikka S, Partanen J, Bellosta P, Coulier F, Basilico C, Jaye M and Alitalo K (1992) Fibroblast growth factor receptor-4 shows novel features in genomic structure, ligand binding and signal transduction. *EMBO J* **11**: 4273–4280

Weiner HL and Swain JL (1989) Acidic fibroblast growth factor mRNA is expressed by cardiac myocytes in culture and the protein is localised to the extracellular matrix. *Proc Natl Acad Sci USA* **86**: 2683–2687

Wolf C, Rouyer N, Lutz Y, Adida C, Loriot M, Bellocq J-P, Chambon P and Bassett P (1993) Stromelysin 3 belongs to a subgroup of proteinases expressed in breast carcinoma fibroblastic cells and possibly implicated in tumor progression. *Proc Natl Acad Sci USA* **90**: 1843–1847

Yamanaka Y, Friess H, Buchler M, Beger HG, Uchida E, Onda M, Kobrin MS and Korc M (1993) Overexpression of acidic and basic fibroblast growth factors in human pancreatic cancer correlates with advanced tumour stage. *Cancer Res* **53**: 5289–5296

Yeoman LC (1993) An autocrine model for cell- and matrix-associated fibroblast growth factor. *Oncol Res* **5**: 489–499

 *© Cancer Research Campaign 1997*